# Selenium Nanoparticles Derived from *Moringa oleifera* Lam. Polysaccharides: Construction, Stability, and In Vitro Antioxidant Activity

**DOI:** 10.3390/foods14060918

**Published:** 2025-03-07

**Authors:** Liang Tao, Chunhua Guan, Zilin Wang, Yue Wang, Quzheng Gesang, Jun Sheng, Jiahe Dai, Yang Tian

**Affiliations:** 1 College of Food Science and Technology, Yunnan Agricultural University, Kunming 650201, China; taowuliang@163.com (L.T.); 13885721152@163.com (C.G.); wangzilin928@163.com (Z.W.); wangyue981117@163.com (Y.W.); gsqz13579@163.com (Q.G.); shengjun_ynau@163.com (J.S.); 2 Engineering Research Center of Development and Utilization of Food and Drug Homologous Resources, Ministry of Education, Yunnan Agricultural University, Kunming 650201, China; 3 Yunnan Key Laboratory of Precision Nutrition and Personalized Food Manufacturing, Yunnan Agricultural University, Kunming 650201, China; 4 Institute of Technology, Pu’er University, Pu’er 665000, China

**Keywords:** selenium nanoparticles, horseradish polysaccharides, stability, antioxidant properties, HepG2 cells

## Abstract

Selenium nanoparticles (SeNPs) have drawn considerable attention to biomedicine, the food industry, and cosmetics due to their strong antioxidant potential and low toxicity. However, their poor stability limits broader applications. A promising strategy to overcome this limitation involves combining SeNPs with polysaccharides. In this study, selenium nanoparticles (MOLP-SeNPs) were synthesized using *Moringa oleifera* Lam. polysaccharide (MOLP) as a stabilizer and dispersant within a redox system comprising sodium selenite and ascorbic acid. The structural characteristics of the synthesized MOLP-SeNPs were analyzed using spectroscopy. Additionally, their thermal and storage stability was evaluated, and their antioxidant activity was explored through simulated digestion in vitro and a HepG2 cell oxidative stress model. The results demonstrated that well-dispersed, zero-valent MOLP-SeNPs showing a mean particle size of 166.58 nm were synthesized successfully through an MOLP-to-sodium selenite ratio of 2.8:3 at pH 7.3 and 35 °C. The MOLP-SeNPs exhibited excellent stability during preparation. In simulated in vitro digestion and H_2_O_2_-induced oxidative stress experiments on HepG2 cells, MOLP-SeNPs displayed strong free radical scavenging capacity while improving antioxidant activity. Cellular experiments deeply revealed that pretreatment with MOLP-SeNPs significantly improved cell viability and provided a pronounced protective effect against oxidative damage. In conclusion, MOLP-SeNPs represent a novel antioxidant with promising applications in food and biomedicine.

## 1. Introduction

As a key trace element, selenium (Se) is an important player in human growth and development, exhibiting anti-inflammatory, antimicrobial, and antioxidant properties [1,2]. Additionally, selenium is a crucial component of glutathione peroxidase, an enzyme protecting cells against the detrimental effects of free radicals during oxidative stress [3]. Selenium cannot be synthesized by human bodies, leading to the necessity of obtaining it from external sources, which mostly exist in the form of inorganic selenium [4]. However, inorganic selenium has low bioavailability and a certain degree of toxicity. Selenium toxicity can occur when intake exceeds the safe limit for the human body [5], which significantly restricts its applications. Therefore, it is essential to develop a high-efficiency selenium supplement with low toxicity. Recently, selenium nanoparticles (SeNPs) have gradually garnered attention, on account of their high bioactivity and low toxicity [6], exhibiting unique functional properties due to their nanoscale size, such as antioxidant [7], antifungal [8], and antitumor [9] effects. Notably, SeNPs are generally unstable, prone to aggregation and precipitation, difficult to store, and susceptible to conversion into gray and black elemental selenium. Furthermore, their bioavailability and biological activity are often compromised due to their surface hydrophobicity and other factors [10]. These limitations hinder the utilization of selenium nanoparticles in the domains of food and biomedicine.

Polysaccharides are biomolecules known for their superior biocompatibility, eco-friendliness, and stability [11,12]. In the food industry, SeNPs are primarily synthesized by reducing sodium selenite to zero-valent selenium, a process facilitated by reducing agents such as ascorbic acid. Following this, natural polysaccharides with bioactive properties are introduced as stabilizers and dispersants, effectively preventing the aggregation and precipitation of SeNPs. Previous research has demonstrated that polysaccharide SeNPs exhibit enhanced stability and bioactivity after the incorporation of *Prunella vulgaris* L. polysaccharide [13], *Usnea longissima* lichen polysaccharide [9], and Heteropolysaccharide isolated from the residue of *Sanghuangporus vaninii* [14] to stabilize the selenium nanoparticles. The use of natural polysaccharides as stabilizers and dispersants for SeNPs to prepare polysaccharide selenium nanoparticles has garnered increasing attention from researchers.

Polysaccharide is a high-quality, plant-derived polysaccharide extracted from the leaf of *Moringa oleifera* Lam., which has extensively applications and significant medicinal implications. *Moringa oleifera* Lam. leaf polysaccharide (MOLP) is composed of various monosaccharides, primarily including galactose, glucose, mannose, arabinose, and xylose. These monosaccharides are linked by glycosidic bonds to form complex polysaccharide chains. Studies have demonstrated that MOLP exhibits strong antioxidant, anti-inflammatory, and hypoglycemic effects [15,16]. MOLP is widely used in a variety of functional foods and pharmaceuticals on account of their low toxicity, high bioactivity and minimal adverse effects [17,18]. However, there is a lack of research on the binding mechanism of selenium with MOLP and their synergistic antioxidant effects.

This study utilized MOLP as a stabilizer and dispersant for SeNPs, preparing *Moringa oleifera* Lam. leaf polysaccharide–selenium nanoparticles (MOLP-SeNPs) by modifying SeNPs. Computer simulations, spectroscopy, and thermodynamics were utilized to elucidate the structural variations of selenium-incorporated Moringa polysaccharide nanoparticles. Furthermore, the antioxidant activity of the nanoparticles was appraised by formulating in vitro models that simulated gastrointestinal digestion and oxidative stress among HepG2 cells. Our research served to lay a good groundwork for the SeNP modification and to expand the application of highly active selenium supplements.

## 2. Materials and Methods

### 2.1. Materials and Reagents

The *Moringa oleifera* Lam. leaf powder was purchased from Dehong Tianyou Technology Development Co., Ltd. (Yunnan, De Hong, China). Na_2_SeO_3_ was supplied by Xi’an Lingfeng Biotechnology Co., Ltd. (Xi’an, China). The ascorbic acid was purchased from the Shanghai Aladdin Biochemical Technology Co., Ltd. (Shanghai, China). HepG2 cells line-SCSP-510 were supplied by the Shanghai Cell Bank of the Chinese Academy of Sciences. (Shanghai, China). The medium (MEM), fetal bovine serum (FBS), and penicillin–streptomycin solution were provided by Wuhan Service Technology Co., Ltd. (Wuhan, China). MTT was provided by Sigma (St. Louis, MO, USA). All other reagents were of analytical grade.

### 2.2. Preparation of MOLP-SeNPs

#### 2.2.1. Extraction of MOLP

MOLP were extracted using the hot water immersion method, as referenced by Sharma et al. [19]. An appropriate amount of *Moringa oleifera* Lam. leaf powder was weighed. The distilled water was supplemented in light of the material–liquid proportion of 1:10 g/mL. The mixed solution was agitated and heated within a water bath at 70 °C for 90 min, before centrifugation in a floor-type low-speed cryo-centrifuge at 4200 rpm for 10 min. The supernatant was gathered, while the solid residue remaining at the bottom of the centrifuge flask was discarded. Distilled water was added again according to the same material-to-liquid proportion of 1:10 (g/mL). The water bath operation was repeated to extract polysaccharides through hot-water leaching a total of three times in total, then the filtrates were integrated. The filtrate was concentrated to 10% of its original volume using rotary evaporation, before 9-volume anhydrous ethanol was added relative to the concentrated filtrate for precipitation. Afterward, the mixed solution was allowed to stand all night. The precipitate was centrifuged at 4200 rpm for 10 min and subsequently freeze-dried.

#### 2.2.2. Single-Factor Experiment

MOLP-SeNPs were synthesized following the method described by Xiao [20]. A 4 mg/mL sodium selenite solution was integrated with a 1 mg/mL MOLP solution at various volume ratios of 1:10, 1:3, 1:2, 1:1, and 4:3. The mixtures were prepared at temperatures of 15, 25, 35, 45, and 55 °C; and at pH levels of 4.0, 6.0, 8.0, 10.0, and 12.0. The solutions were stirred overnight. Following this, an equal volume of 4 mg/mL ascorbic acid solution was incorporated into the mixture and agitated at normal temperature for 6 h to obtain MOLP-SeNPs. The resulting MOLP-SeNPs solution was separated by dialysis through a 3500 Da molecular weight cutoff dialysis bag (Spectrum Medical Industries, Inc., Fairfax, VA, USA) for 2 days, with distilled water being replaced every 4 h. Then, the MOLP-SeNPs were freeze-dried in vacuum conditions and preserved at −20 °C for future use as needed.

#### 2.2.3. Response Surface Methodology (RSM) Optimization

Box–Behnken Design (BBD) is a specific type of experimental design within RSM that enables efficient exploration of optimal conditions in multifactorial systems through a limited number of experiments [21]. The process was optimized using a BBD according to one-factor experimental results. A 3-factor, 3-level assay was implemented to refine the fabrication procedure of MOLP-SeNPs using the BBD design, with volume ratio, pH, and temperature as response surface factors. Moreover, the particle size of MOLP-SeNPs was regarded as the response variable (*Y*). Table 1 presents the factor levels. In Table 2, the experimental data were construed using multiple regression analysis through Design-Expert. The functionality provided in Design-Expert version 13 was utilized for this purpose.

### 2.3. Interaction Between Moringa Oleifera Polysaccharide and Selenium

#### 2.3.1. Molecular Docking

The SDF (Structure Data File) for selenium ions was obtained by searching the PubChem website. Molecular docking between the identified monosaccharides and selenium particles was performed through Hex software version 8.0.0. Affinity (grid) maps were produced showing a spacing of 0.375 Å. Finally, the optimum conformation was outputted in accordance with the grid score (kcal/mol).

#### 2.3.2. Quantum Chemistry Calculations

The molecules were optimized using the Material Studio 2023 software, specifically employing the DMol3 module for Density Functional Theory (DFT) calculations. The Generalized Gradient Approximation (GGA) with the Perdew–Burke–Ernzerhof (PBE) functional was adopted to characterize the exchange-relevance interactions, and the employed basis set was the double-numerical plus polarization (DNP) basis set. Upon achieving the optimized structures, the Discovery Studio 3.5 software package (Merrys Software, Inc., San Diego, CA, USA) was used, followed by transferring the structures back to the Gaussian 09 package for further processing. This process included structural optimization, energy calculation, and the removal of imaginary frequencies through frequency calculations. Thermodynamic adjustments were applied to the optimized sugar–Se conformations to estimate the energy of the sugar–selenium complex at 298 K.

### 2.4. Structural Characterization and Analysis

#### 2.4.1. Monosaccharide Composition

A mass of 5 mg of the sample was accurately weighed and placed into an ampoule. Subsequently, 2 mL of 3M trifluoroacetic acid (TFA) was incorporated for hydrolysis, which was conducted at 120 °C for 3 h. Afterward, the mixture was dehydrated under nitrogen gas. Next, 5 milliliters of distilled water was supplemented. The mixture was thoroughly vortexed with centrifugation at 12,000 revolutions per minute for 5 min. The supernatant was gathered for ion chromatography analysis. The chromatographic conditions were listed below: Analysis was performed through an ion chromatograph (ICS5000, Thermo Fisher, Waltham, MA, USA) with a Dionex CarbopacTM PA10 (4 × 250 mm) column. The mobile phases were: A: H_2_O; B: 500 mM NaOH & 50 mM NaOAc; C: 20 mM. The flow rate was set at 1.0 mL/min, the injection volume was 25 µL, the column temperature remained at 30 °C, and the detection was implemented using an electrochemical detector.

#### 2.4.2. Dynamic Light Scattering

The MOLP-SeNPs were analyzed for particle size. The polydispersity index (PDI) was construed through a Multi-Angle High Sensitivity Zeta Potential Analyzer (NanoBrook Omni, Brookhaven Instruments, Nashua, NH, USA). The refractive indices of the MOLP-SeNPs solution and water were 1.520 and 1.330, separately. The total measurements were executed at 25 °C.

#### 2.4.3. Fourier Infrared Spectroscopy (FT-IR)

Freeze-dried specimens of MOLP-SeNPs (2 mg each) were mixed and loaded with 300 mg dried potassium bromide (KBr) and loaded into a FT-IR spectrometer (iS5, Thermo Fisher, Waltham, MA, USA). All FTIR spectra from 4000 to 400 cm^−1^ were noted. Measurements were conducted in a dry environment at room temperature.

#### 2.4.4. X-Ray Diffraction (XRD)

The XRD spectra of specimens were gauged through an X-ray diffractometer (SmartLab, Rigaku, Japan), employing a powder XRD technique. Such samples were scanned throughout a 2θ varying between 10 to 80° using Cu Kα (λ = 1.54 Å) as a source of radiation. The measurement was conducted at 40 kV (operating voltage).

#### 2.4.5. Fluorescence Spectroscopy (FS)

MOLP and MOLP-SeNPs were decomposed in deionized water at a concentration of 0.1 mg/mL. The FS of both MOLP and MOLP-SeNPs were documented through a fluorescence spectrophotometer (F-2700, Hitachi Ltd., Tokyo, Japan) with an excitation wavelength of 295 nm and emission wavelengths varying from 300 to 500 nm.

#### 2.4.6. X-Ray Diffraction Photoelectron Spectroscopy (XPS)

The crystal morphology of MOLP-SeNPs was gauged using an X-ray diffractometer (ESCALAB 250×, Thermo Fisher, USA). The 2θ angle was scanned between 5 to 95° at a rate of 5°/min, and the operating voltage and current were initialized to 40 kV and 40 mA, separately. A monochromatic Alka source (energy 1486.6 eV) was employed as the excitation source.

#### 2.4.7. Scanning Electron Microscopy (SEM)

The microscopic composition of MOLP and MOLP-SeNPs were researched via a scanning electron microscope (SEM-7800F, JEOL Ltd., Tokyo, Japan). Through the conductive adhesive binder, the dried powders were immobilized on a specimen holder separately. The samples unadhered were blown off with a wash ball, after which a 15 nm-thick layer of gold powder was applied to the surface of specimens. A current of 15 mA was applied for 90 s. Such samples were examined at an acceleration voltage of 5 kV.

### 2.5. Stability Measurements of MOLP-SeNPs

#### 2.5.1. Differential Scanning Calorimetry (DSC) and Thermogravimetry (TG)

The thermal stability of MOLP-SeNPs was appraised through a simultaneous thermal analyzer (STA-8000, PerkinElmer, Waltham, MA, USA). Approximately 2–4 mg MOLP-SeNPs were placed in an alumina crucible with a perforated lid. The gas flow rate of nitrogen was set at 20 mL/min, with the heating rate reaching 10 °C/min. The measurement temperature ranged from 30 to 800 °C.

#### 2.5.2. Storage Stability

The storage stability of MOLP-SeNPs was assessed via gauging the particle size of MOLP-SeNPs preserved at 4 and 25 °C over periods of 0, 7, 14, 21, 28, and 35 days.

### 2.6. Gastrointestinal Digestion and Antioxidant Studies

#### 2.6.1. Simulated Oral, Gastric, and Intestinal Digestion in Vitro

The method of digestion in vitro was slightly modified from that described by Paula [22] and Jia [23]; in brief, 20 mg of MOLP or MOLP-SeNPs chelate was decomposed within 10 mL distilled water, preheated to 37 °C within a water bath, and subsequently blended with 10 mL artificial saliva, which was modulated to a pH of 6.8. The mixed solution was oscillated at 100 rpm for 10 min at 37 °C. Following this, the pH was modulated to 1.0 using 1 M hydrochloric acid, and 3.75 μL simulated gastric digest was taken for 90 min. Afterward, another 3.75 μL simulated gastric digest was taken and digested for extra 90 min. Following gastric digestion, the pH was modulated to 7.5 using 1 M NaHCO_3_, and 13.125 μL of simulated intestinal digest was supplemented. The mixture was then shaken at 37 °C for 120 min. Samples from each phase of digestion were gathered with centrifugation at 8000 rpm at 4 °C for 10 min. The supernatant was carefully eliminated and preserved at −80 °C.

#### 2.6.2. ABTS^•^+ (2,2′-Azino-bis(3-ethylbenzothiazoline-6-sulfonate) Radical Cation) Clearance Viability

The ABTS^•^+ scavenging activity of digested MOLP-SeNPs extracts was evaluated using a method adapted from Yao [24] with slight alternations. An ABTS solution was formulated by combining 7 mmol/L ABTS stock solution showing an equal volume of 2.45 mmol/L potassium persulfate solution. This mixture was brooded in darkness for 12 h to 16 h and subsequently diluted with methanol till the absorbance at 734 nm reached 0.70 ± 0.02. After that, 0.2 mL of the sample solution was integrated with 3.8 mL of the ABTS solution. Following 6 min incubation, the absorbance at 734 nm was gauged. A methanol solution served as a blank control in place of the sample.

#### 2.6.3. •OH Scavenging Ability

The capacity to scavenge hydroxyl radicals (•OH) was gently modified following the approach by Jia [23]. This procedure involved sequentially adding 1 mL of the sample, 6 mmol/L ferrous sulfate, 6 mmol/L hydrogen peroxide, and 6 mmol/L salicylic acid–ethanol solution. The mixed solution was then agitation and enabled to react at 37 °C for 30 min, following which the absorbance was determined at 510 nm. Distilled water acted as a blank control replacing the sample.

#### 2.6.4. Modeling of H_2_O_2_-Induced Oxidative Stress

HepG2 cells were nurtured within the DMEM substrate added with 10% fetal bovine serum and 1% penicillin–streptomycin solution. The cells were brooded at 37 °C in 5% CO_2_ and managed with diverse concentrations of H_2_O_2_ (0.05, 0.1, 0.2, 0.4, 0.6, and 0.8 mmol/L) for 4 h to induce oxidative stress. Untreated cells served as controls.

#### 2.6.5. Cell Viability Assay

The influences of MOLP-SeNPs and H_2_O_2_ on the activity of HepG2 cells were assessed using the MTT assay, as described earlier. For the proliferation assays, cells were processed with changing concentrations of MOLP-SeNPs (5.0–40.0 μg/mL) for 24 h. For the protective experiments, cells were preprocessed with the identical concentrations of MOLP-SeNPs for 24 h, before treated with 0.6 mM H_2_O_2_ for 4 h. Untreated cells served as controls. Then, such cells were brooded with 200 μL fresh substrate with MTT (5.0 mg/mL) for 4 h. Subsequently, 150 μL of DMSO was supplemented in place of the culture medium. The 96-well plate was shaken, with the intention of preventing DMSO crystals from forming. Ultimately, the absorbance at 570 nm was determined through an enzyme labeling instrument (Multiskan GO, Thermo Fisher, USA). Cell viability was figured out as follows:

$$\mathrm{Cell}\;\mathrm{viability}\;(\%)=(A_{T}/A_{C})*100$$

Here, *A_T_* represents the absorbance of the experimental group and *A_C_* represents the absorbance of controls (without treatment).

### 2.7. Statistical Analysis

Graph processing was conducted through Origin 2024, GraphPad 10, and Design-Expert 13. Statistical analysis was executed through SPSS 27 software. Statistical significance was measured via Duncan’s Multiple Range Test through one-way ANOVA, with *p* < 0.05 holding statistical significance. Our measurements were conducted in triplicate, with all data denoted as mean ± standard deviation (*n* ≥ 3).

## 3. Results and Discussion

### 3.1. Preparation and Process Optimization of MOLP-SeNPs

#### 3.1.1. Optimization of Conditions for the Preparation of MOLP-SeNPs in Single-Factor Experiment

**Figure 1:**
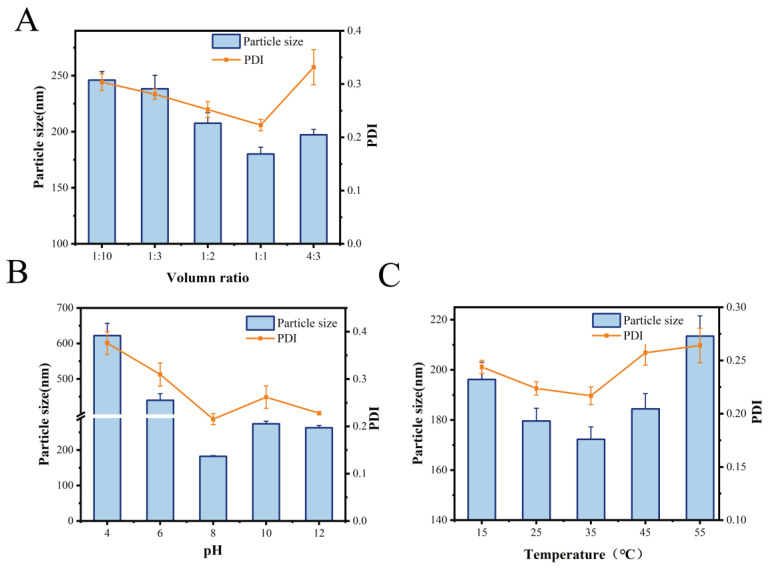
Particle size and PDI of MOLP-SeNPs at discrepant volume ratios (**A**), pH (**B**), and temperature (**C**).

Particle size is an essential parameter for evaluating the degree of aggregation in MOLP-SeNPs [25]. Similarly, the PDI serves as an essential metric for assessing the stability of the polymer system [26]. Figure 1A illustrates the effect of varying MOLP/Na_2_SeO_3_ volume ratios on the particle size and PDI of the synthesized MOLP-SeNPs. As the MOLP volume rose, the particle size and corresponding PDI initially fell and then increased. The smallest values were recorded at a 1:1 volume ratio, with a diameter of 180.15 ± 10.51 nm and a PDI of 0.22. When the MOLP/Na_2_SeO_3_ ratio ranged from 1:10 to 1:1, both particle size and PDI gradually decreased, likely due to the sufficient reaction between sodium selenite and MOLP. However, at a MOLP/Na_2_SeO_3_ ratio of 4:3, further increases in MOLP volume led to a rise in both particle size and PDI, possibly because excess MOLP failed to effectively bind with selenium. This resulted in nanoparticle aggregation, increasing particle size and reducing system stability.

The effect of pH on particle size and PDI, as displayed in Figure 1B, revealed an obvious decrease in particle size from 450 ± 18.38 to 182.25 ± 13.39 nm as pH varied between 4.0 and 8.0. However, since pH continued to rise from 8.0 to 12.0, both particle size and PDI progressively increased. This behavior may be attributed to protonation under weakly acidic conditions, which weakens electrostatic interactions between MOLP and selenium, leading to nanoparticle aggregation. Conversely, under alkaline conditions, some selenium ions may precipitate with hydroxide ions (OH^−^), reducing the available selenium and causing slight turbidity in the reaction system. Similar findings have been reported for Umbelliferae polysaccharide-coated SeNPs [27] and Astragalus polysaccharide–selenium nanocomplexes [28].

Reaction temperature is another crucial factor influencing nanoparticle size. As illustrated in Figure 1C, particle size was minimized at 35 °C, showing a diameter of 172.29 ± 11.72 nm and a PDI of 0.217. Findings suggest that temperature is a key player in synthesizing MOLP-SeNPs. Higher temperatures enhance the chelation between polysaccharides and selenium; however, excessively high temperatures may disrupt this process. When the chelation rate is lower than the dissociation rate, nanoparticle formation is negatively affected. To sum up, the optimal conditions for synthesizing MOLP-SeNPs, based on single-factor experiments, were a 1:1 MOLP-to-Na_2_SeO_3_ volume ratio, a pH of 8, and a reaction temperature of 35 °C.

#### 3.1.2. Optimization of Preparation Conditions for MOLP-SeNPs Using RSM

RSM for experimental optimization is an effective experimental design approach. It involves constructing mathematical models to describe the relationship between experimental factors and experimental outcomes, and solving optimization problems to identify optimal experimental conditions [29]. Based on the results of one-way experiments, 17 optimization experiments were conducted with particle size (*Y*) as the response variable and volume ratio (*A*), pH (*B*), and temperature (*C*) as three factors (Table 1 and Table 2). Additionally, the data was fitted to a quadratic polynomial regression equation, bringing about the listed polynomial equation for particle size (*Y*):

*Y* = 180.37 − 20.28*A* + 41.02*B* + 16.15*C* + 21.82*AB* + 4.1*AC* + 43.26*BC* + 72.67*A*^2^ + 59.99*B*^2^ + 72.86 *C*^2^

ANOVA was utilized to rate the effectiveness of the quadratic polynomial model. Table 3 presents the outcomes. The raised regression equation fitted the experimental data showing low error and the independent variables remarkably affected the results [30], as shown by the regression test at *p* < 0.0001 and the regression model at *p* = 0.5131 > 0.05. The coefficient of determination *R*^2^ = 0.9829, the coefficient of adjustment *R*^2^adj = 0.9610, the Adeq precision of 19.804, and the coefficient of variation *CV* of 5.54% indicate that this test is credible and the research results meet the requirements of model reproducibility.

**Figure 2:**
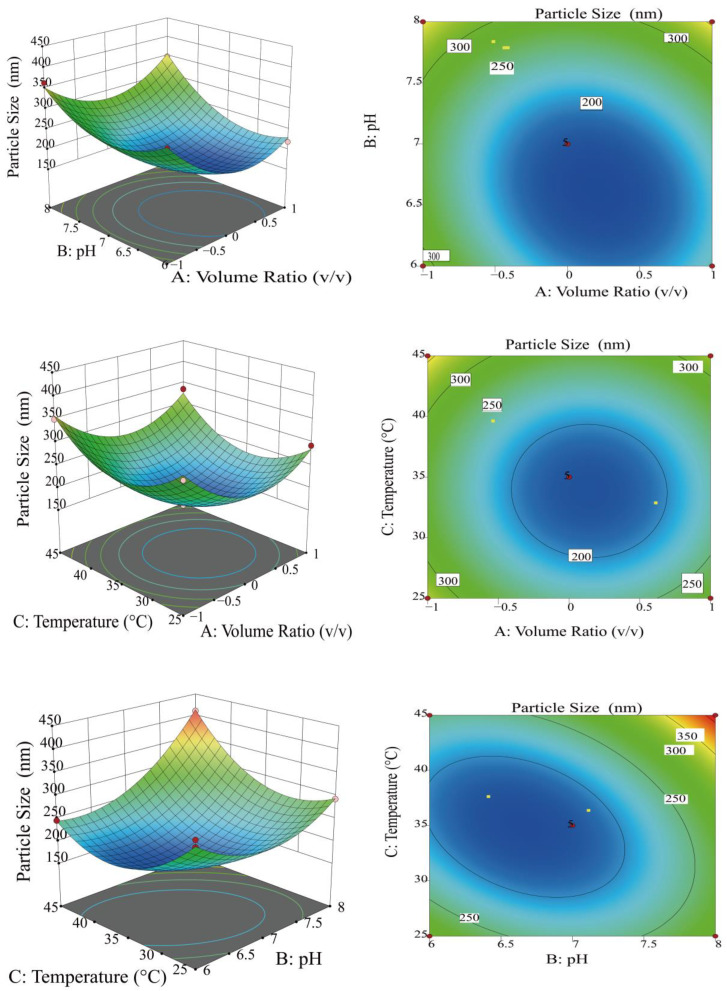
Response surface plots and contour plots of effect of interaction of factors on particle size.

Figure 2 exhibits 3D surface response maps and contour plots, which exhibit saddle-shaped and elliptical contours, indicating a significant interaction between the two factors [31]. As the *BC* slope increased, the interaction became exceedingly remarkable (*p* < 0.01), while the AB interaction became marked (*p* < 0.05). In contrast, the *AC* interaction was not remarkable (*p* > 0.05), and the BC interaction was excessively remarkable (*p* < 0.01). Based on p-values, the relative importance of each factor’s effect on particle size was established as follows: pH (*B*) > volume ratio (*A*) > temperature (*C*). The magnitude of the effect on particle size was ranked in the order of *BC* > *AB* > *AC*. The application of RSM identified the optimal chelation conditions as a volumetric ratio of 2.79:3 for horseradish polysaccharides to sodium selenite, a pH of 7.25, and a temperature of 34.95 °C. Results were derived from the study and were utilized to measure the optimal chelating conditions.

To guarantee the credibility of the optimization outcomes, the parameters were modulated according to laboratory operations. The optimal process parameters for the preparation of selenium nanoparticles from Moringa polysaccharides were determined to be a volume ratio of 2.8:3, a pH of 7.3, and a temperature of 35°C. Through such parameters, a particle size of 166.58 ± 2.63 nm was achieved after conducting three parallel experiments. This result reflects a deviation of 2.39% from the predicted total particle size of 170.66 nm, indicating that the model demonstrates high reliability and predictive accuracy.

### 3.2. Identification of Monosaccharide Composition and Interaction Between Monosaccharides and Se

#### 3.2.1. Monosaccharide Composition

**Figure 3:**
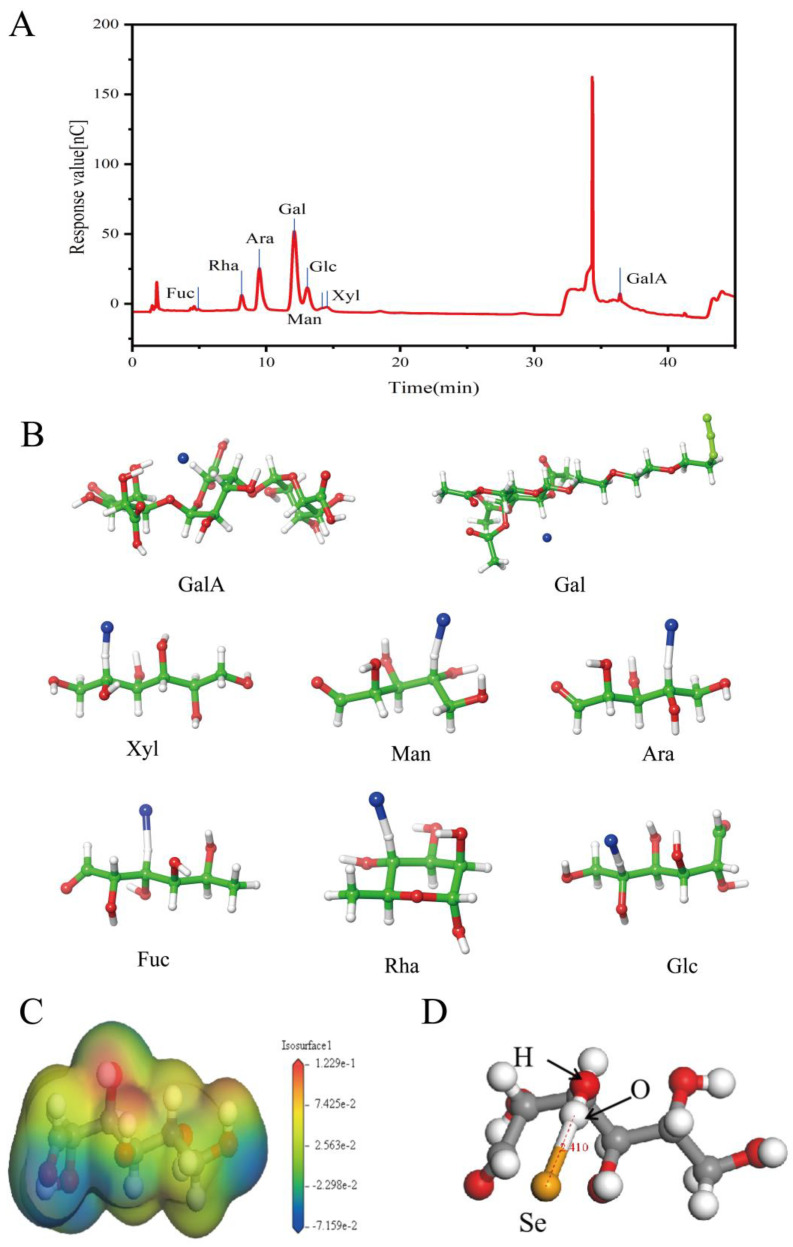
Ion chromatography (**A**). Molecular docking results (**B**). Electron density distribution map of Glc-Se complex. Color bar suggests corresponding charge value of each region (**C**). Converged structure calculated through Gaussian (using ball-and-stick model) features yellow spheres representing Se. Dashed lines indicate relative position of Se to oxygen atoms, with a distance of 2.410 Å. (**D**).

In this study, the extraction yield of MOLP was 11.26 ± 0.52%. Figure 3A presents the monosaccharide structure of *Moringa oleifera* polysaccharides. The results indicate that polysaccharides extracted from *Moringa oleifera* leaves comprise eight monosaccharides: arabinose (Ara), fucose (Fuc), galactose (Gal), galacturonic acid (GalA), glucose (Glc), rhamnose (Rha), mannose (Man), and xylose (Xyl). Their respective molar percentages are 20.79, 0.56, 49.60, 7.29, 9.89, 7.89, 2.11, and 1.88%, respectively. Among these, arabinose and galactose are the dominant monosaccharides, which corresponds to the discoveries of Husien et al. [32], though some variations in their proportions were observed.

#### 3.2.2. Molecular Docking

Molecular docking simulations provide theoretical validation of the experimental results and enable a more precise analysis of the interactions between MOLP and Se during the binding process. As shown in Figure 3B, the polysaccharides from *Moringa oleifera* leaves consist of eight monosaccharides: Ara, Fuc, Gal, GalA, Glc, Rha, Man, and Xyl. Molecular docking was performed between each of these monosaccharides and Se individually. The docking results, presented in Figure 3A, show binding energies of −33.25, −31.42, −33.39, −40.58, −32.16, −33.69, −30.46, and −40.19 kcal/mol for Ara, Fuc, Gal, Glc, Man, Rha, Xyl, and GalA with Se, respectively. Since all binding energies are negative, the interactions are thermodynamically favorable. If a binding energy is lower, it will indicate a more stable conformation [21], suggesting that the binding interaction between MOLP and Se is highly stable. Additionally, the binding sites of Se on each monosaccharide were found to be -CH functional groups.

#### 3.2.3. DFT

While molecular docking provides preliminary predictions of the sugar–Se binding mode, it cannot determine the exact spatial structure of the sugar–Se complex. Since Glc exhibited the most significant negative binding energy (−40.58 kcal/mol) in molecular docking, we employed quantum chemical methods based on DFT to calculate its precise spatial structure in complex with Se. Figure 3C presents the electron cloud density of Glc, while Figure 3D displays the optimized structure obtained from Gaussian calculations. The results indicate that Se can stably bind to the -OH group, reaching equilibrium with the oxygen atom at a relative distance of 2.410 Å. The calculated binding energy of the complex is −10.47 kcal/mol, confirming that Glc forms a stable structure with Se. However, these results deviate from those obtained through molecular docking, likely due to fundamental methodological differences. Molecular docking relies on computational chemistry to quantify interactions between small and large molecules, whereas DFT provides a quantum mechanical perspective on molecular interactions [33]. In this study, Glc demonstrated a stable spatial structure, and its binding with Se was energetically feasible. The findings suggest that Glc effectively interacts with Se through both -CH and -OH groups, forming a stable complex.

### 3.3. Structural Characterization of MOLP-SeNPs

#### 3.3.1. FT-IR

**Figure 4:**
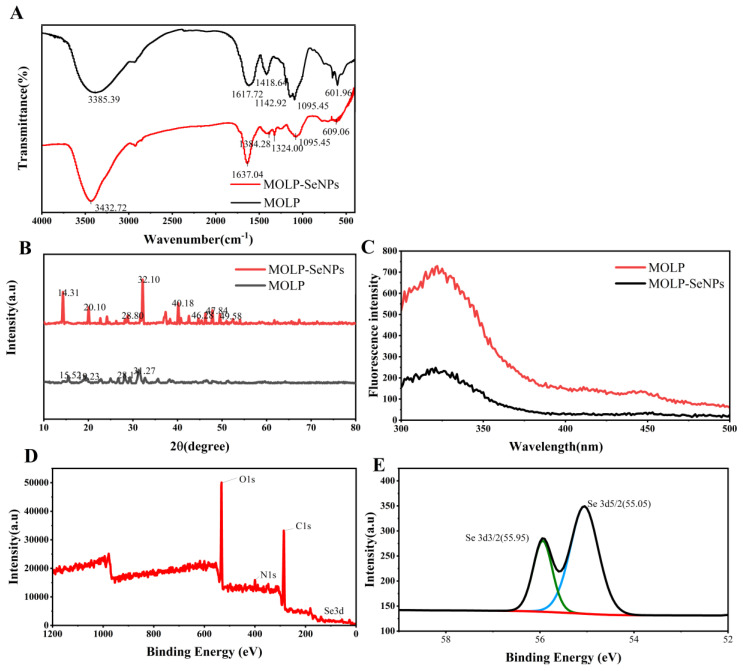
Structural characterization of MOLP and MOLP-SeNPs. FT-IR spectra (**A**). XRD spectra (**B**). FS within Wavelength Range of 300–500 nm (**C**). General scan spectrum (**D**) and Se 3d region scan spectrum (**E**) in XPS of MOLP-SeNPs.

The binding mechanism between MOLP and Se was investigated using FT-IR spectroscopy, as shown in Figure 4A. In the amide A band, MOLP exhibited a characteristic peak at 3385.39 cm^−1^ with strong absorption, ascribed to tensile vibrations of O-H and N-H bonds [34]. By contrast, the peak shifted to 3432.72 cm^−1^ in MOLP-SeNPs, suggesting that Se interacts with the polysaccharide via the -OH group [35]. The amide I band, associated with the secondary structure of polysaccharides, displayed a characteristic peak at 1616.71 cm^−1^ in MOLP due to C-O stretching vibrations. Following Se chelation, this peak red-shifted to 1637.04 cm^−1^ in MOLP-SeNPs, likely due to hydrogen bond formation, which reduced the electron cloud density of the C-O bond and triggered the observed spectral shift [36]. Additionally, the characteristic peak of MOLP at 1418.64 cm^−1^, attributed to C-H bending vibrations, weakened and shifted to 1384.28 cm^−1^ after chelation with sodium selenite. This shift suggests that Se binds to MOLP at the -CH functional group [37]. In conclusion, the observed peak shifts in MOLP-SeNPs indicate that the primary selenium binding sites on MOLP involve -OH, C-O, and -CH functional groups.

#### 3.3.2. XRD Spectral Analysis

XRD spectroscopy is a powerful technique for characterizing crystal structures and their variation patterns [38]. The XRD results for MOLP and MOLP-SeNPs are presented in Figure 4B. In the MOLP group, four major but dispersed diffraction peaks were observed at approximately 15.52, 19.23, 28.17 and 31.27°, all exhibiting relatively weak intensities. Following selenium incorporation, these peaks shifted to 14.31, 20.10, 28.80, and 32.10°, respectively, accompanied by noticeable changes in peak width and height. Furthermore, the diffraction pattern of MOLP-SeNPs revealed the emergence of sharp new peaks at 40.18, 46.28, 47.84, and 49.58°, along with several weaker peaks. Such changes in the initial peaks, coupled with the appearance of new peaks, suggest the formation of novel crystalline structures resulting from the chelation of polysaccharides with selenium [24]. Additionally, the crystallinity of MOLP-SeNPs increased significantly compared to MOLP alone, illustrating that new crystal phases form. This confirms the outstanding chelation of MOLP with selenium, causing the formation of MOLP-SeNPs.

#### 3.3.3. FS

The fluorescence spectra (FS) of MOLP and MOLP-SeNPs are indicated in Figure 4C. Findings illuminate that selenium binding to MOLP significantly reduces fluorescence intensity. In MOLP-SeNPs, selenium acts as a quencher, forming stable, non-luminescent complexes with MOLP molecules. This quenching occurs before the formation of the excited state, leading to a decrease in fluorescence intensity [39]. The reduction in fluorescence may be attributed to the folding and accumulation of some sugar backbones during the chelation process with Se [40,41]. Additionally, the introduction of mineral ions can sometimes induce a fluorescence burst in the sample, a phenomenon similar to that observed by Lin et al. [42] in peptide–calcium complexes.

#### 3.3.4. XPS Spectral Analysis

Selenium has four different oxidation states (−2, 0, +4, +6), among which zero-valent SeNPs shows lower toxicity and higher bioactivity [43,44]. To confirm the zero-valent state of MOLP-SeNPs, we conducted elemental analysis and Se 3d spectroscopic scans. The overall scan (Figure 4D) reveals strong C1s, O1s, and N1s signals, primarily originating in MOLP, while the Se signal appears weak. This may be due to two factors. First, X-ray photoelectron spectroscopy (XPS) detects surface elements via bombarding the sample with X-rays, which release photoelectrons from the internal atomic shells. If Se is encapsulated within the MOLP structure, its signal may be suppressed [45]. Second, the detected signal intensity is roughly proportional to element concentration, suggesting that the Se content in MOLP-SeNPs is relatively low. Among the peaks, the binding energies of Se 3d 5/2 and Se 3d 3/2 were detected at 55.05 eV and 55.95 eV, separately, corresponding to the expected values for Se(0) [46]. Compared to the standard spectrum of SeNPs, these peaks ranged slightly from 55.1 eV and 56.05 eV to 55.05 eV and 55.95 eV, indicating an interaction between MOLP and Se.

#### 3.3.5. SEM

**Figure 5:**
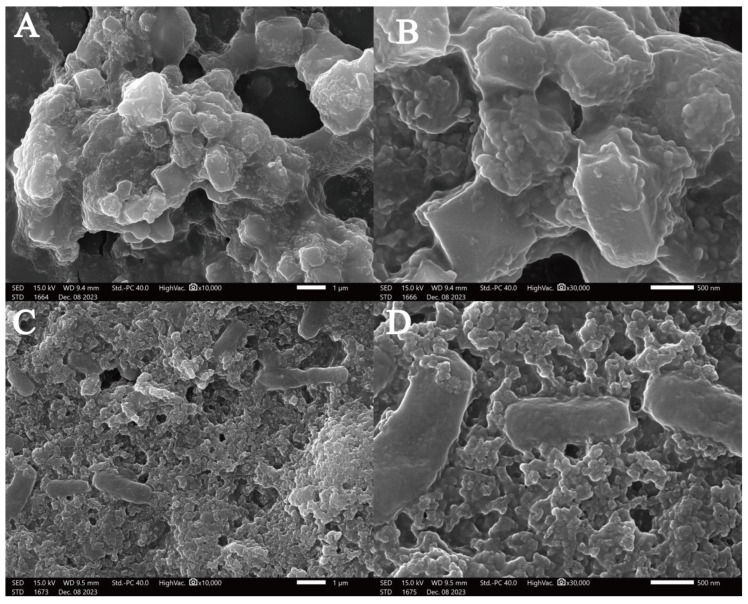
Scanning electron micrographs of MOLP (**A**,**B**) and MOLP-SeNPs (**C**,**D**) at discrepant magnifications (**A**,**C**) ×10,000; (**B**,**D**) ×30,000.

Scanning electron microscopy (SEM) was employed to detected the MOLP microstructure (Figure 5A,B) and MOLP-SeNP chelates (Figure 5C,D) at different magnifications. As shown in Figure 4, MOLP exhibits a microstructure with cavities of varying sizes, composed of particles with different diameters and a relatively smooth surface. After chelation with Se, the resulting MOLP-SeNP chelates displayed a distinct porous and wrinkled surface structure, with no visible agglomeration. The rough and dense surfaces suggest that selenium incorporation significantly affects the aggregation behavior of MOLP. It is speculated that MOLP experiences folding and accumulation upon interaction with Se, primarily via ionic and coordination bonds [47], which corresponds to the XRD results of MOLP-SeNPs.

### 3.4. Stabilization of Selenium Nanoparticles of Polysaccharides from Chorizo Wood

#### 3.4.1. TG-DSC

**Figure 6:**
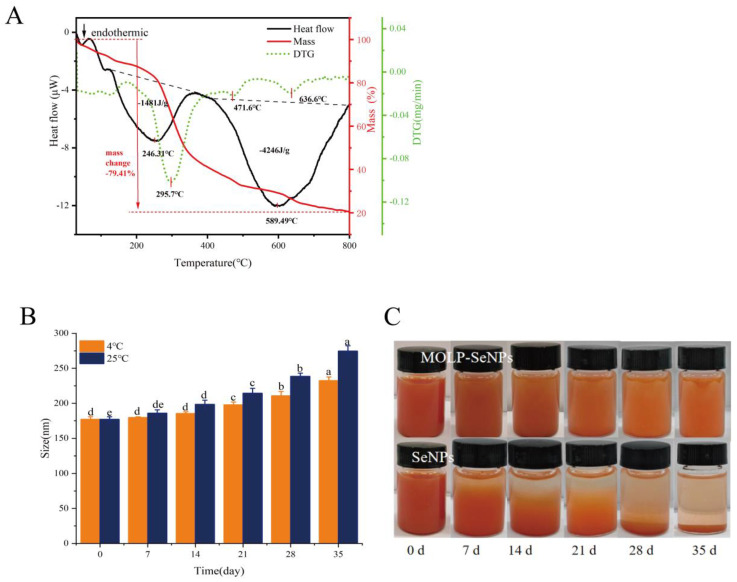
TG/DSC curves of MOLP-SeNPs (**A**). Average diameters of MOLP-SeNPs placed at 4 °C and 25 °C in darkness for 35 days (**B**). Photographs of MOLP-SeNPs and SeNPs preserved at 25 °C in the dark for 0–35 days (**C**). Values showing discrepant letters in the graphs indicate remarkable discrepancies (*p* < 0.05).

To validate the thermal stability of MOLP-SeNPs, TG-DSC curves were recorded for MOLP-SeNPs in a nitrogen atmosphere at temperatures between 30 to 800 °C. As illustrated in Figure 6A, the TG curves indicate that the mass of the samples gradually decreases with increasing temperature, which was linked to the stability of the polysaccharide selenium nanoparticles [48]. The mass loss of MOLP-SeNPs occurred in three primary stages. The initial mass loss between 50 and 200 °C is largely ascribed to water loss, which was positioned outside the polysaccharide backbone and was lost easily. As the temperature surpassed 200 °C, the polysaccharide backbone started to undergo pyrolysis. The TG curve reveals the fastest rate of mass loss at 295.7 °C, where almost all the moisture in the polysaccharide molecules is lost, and partial pyrolysis of the polysaccharide backbone begins [49]. In the DSC curve, two distinct endothermic peaks are observed. The first is an endothermic peak at 246.3 °C showing an endothermic heat of 1481 J/g, whereas the second endothermic peak occurs at 589.5 °C showing an endothermic heat of 4246 J/g. The higher endothermic heat is chiefly ascribed to the higher bond energy of chemical bonds formed in the chelate complex, resulting in a stable structure that requires a higher temperature and more energy for bond dissociation [50]. These findings indicate that MOLP serves as an excellent carrier for SeNPs.

#### 3.4.2. Storage Stability Analysis

Addressing the storage stability of SeNPs is critical for enhancing their application. Figure 6B illustrates the time-dependent change in the average diameter of MOLP-SeNPs in water solution at 4 and 25 °C. At 4 °C, the particle size of MOLP-SeNPs grew as the storage time grew. After 21 days, the particle size prominently rose to 197.73 nm. After 35 days, it rose from 177.09 ± 3.75 to 232.43 ± 5.21 nm. In contrast, at 25 °C, the particle size rose moderately over 7 days, from 177.09 ± 3.75 to 185.99 ± 4.63 nm. After 35 days of storage at 25 °C, the particle size reached 274.71 ± 7.57 nm. These results suggest that MOLP-SeNPs are more stable when stored under refrigerated temperatures and light-avoidance conditions. As shown in Figure 6C, the MOLP-SeNP solution remains transparent with minimal precipitation after storage at 25 °C for 35 days, retaining its characteristic orange-red color. This indicates that the selenium in the solution remains in the zero-valent state, and the solution is relatively stable. In contrast, a freshly prepared SeNP aqueous solution appears turbid due to the aggregation of selenium particles. Following 35 days of storage at 25 °C, the SeNPs nearly completely precipitated at the bottom, with accumulated elemental selenium suspended within the solution. These findings correspond to research on selenium nanoparticles modified with polysaccharides from *Gracilaria lemaneiformis* [49]. The results highlight that MOLP is a key player in the formation and stability of SeNPs.

### 3.5. Antioxidant Activity of MOLP-SeNPs

#### 3.5.1. In Vitro Analysis of Antioxidant Activity After Simulated Digestion

**Figure 7:**
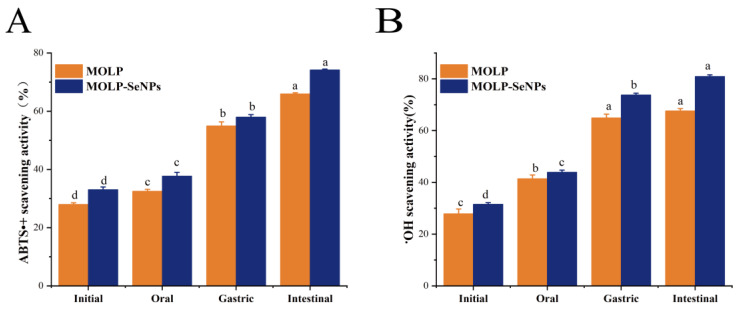
Changes in antioxidant activity during simulated digestion of MOLP-SeNPs and MOLP. (**A**) ABTS^•^+ radical scavenging; (**B**) •OH radical scavenging activity. Values showing discrepant letters in the graphs indicate remarkable discrepancies (*p* < 0.05).

The free radical scavenging activities of ABTS^•^+ and •OH in MOLP and MOLP-SeNPs were evaluated at four digestion phases: undigested, oral, gastric, and intestinal. The changes in ABTS^•^+ scavenging activities of MOLP and MOLP-SeNPs are indicated in Figure 7A. The ABTS^•^+ scavenging activity of MOLP-SeNPs remarkably exceeded that of MOLP. The ABTS^•^+ scavenging activities at the four digestion stages for MOLP-SeNPs were 33.08, 37.72, 58.00, and 74.22%, separately. Relative to the undigested phase, the ABTS^•^+ scavenging activity of both MOLP and MOLP-SeNPs increased sequentially after oral and gastrointestinal digestion, with the highest scavenging activity observed during the intestinal digestion stage (65.96% for MOLP and 74.22% for MOLP-SeNPs). Similarly, the •OH scavenging activities of MOLP-SeNPs (Figure 7B) at the four stages were 32.12, 44.61, 74.60, and 81.75%, respectively, mirroring the ABTS^•^+ results. Compared to MOLP, MOLP-SeNPs exhibited enhanced ABTS^•^+ and •OH scavenging capabilities, consistent with the antioxidant activity results observed for abalone visceral peptides-selenium complexes [23]. This may be attributed to the intrinsic strong antioxidant activity of MOLP. Se, as a potent antioxidant, effectively scavenges free radicals [51]. Consequently, the chelation of MOLP with Se results in the formation of a complex with enhanced antioxidant activity. Overall, the ABTS^•^+ and •OH scavenging capacities of MOLP and MOLP-SeNPs were relatively unaffected by oral digestion but significantly influenced by gastrointestinal digestion. This can be primarily attributed to the enzymatic degradation of polysaccharides by digestive enzymes, like trypsin, pancreatin, pepsin, and bile acids. This degradation exposes more antioxidant active sites and provides additional reactive sites for radicals [52], making the digestive products better interact with ABTS^•^+ and •OH, significantly enhancing their antioxidant activity. These findings illuminate that the formation of MOLP-SeNPs greatly improves the antioxidant activity of SeNPs during digestion, with the antioxidant activity during the intestinal phase reaching nearly 80%. This highlights the potential of MOLP-SeNPs as a bioactive selenium supplement for addressing intestinal oxidative stress diseases.

#### 3.5.2. Effect of H_2_O_2_ and MOLP-SeNPs on HepG2 Cell Viability

**Figure 8:**
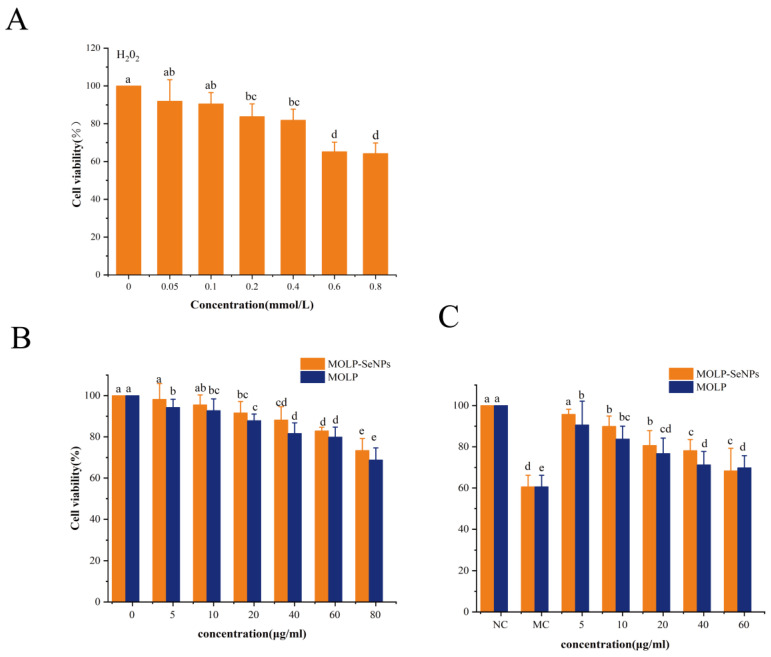
Effects of discrepant concentrations of H_2_O_2_ (**A**) and MOLP-SeNPs on viability of HepG2 cells (**B**). Protective effects of MOLP-SeNPs and MOLP on H_2_O_2_ (0.6 µM)-induced HepG2 cells (**C**). Values showing discrepant letters hold remarkable discrepancies (*p* < 0.05).

Hydrogen peroxide (H_2_O_2_) is a significant reactive oxygen species (ROS) that could induce oxidative damage through cell membranes [53,54]. In this study, H_2_O_2_ was used to construct a model of oxidative damage among HepG2 cells to delve into the protective effect of MOLP-SeNPs on these cells. To gauge the appropriate concentration of H_2_O_2_ for triggering oxidative damage, cell activity was determined by the MTT experiment. As displayed in Figure 8A, cell activity was remarkably (*p* < 0.05) reduced by H_2_O_2_ at concentrations between 0.05 and 0.8 mmol/L relative to controls. In contrast, no remarkable discrepancies occurred in cell activity between the 0.6-0.8 mmol/L H_2_O_2_ treatments. Hence, the appropriate concentration of H_2_O_2_ for inducing oxidative damage was determined to be 0.6 mmol/L in this study.

Both MOLP-SeNPs and MOLP (5.0–80.0 μg/mL) exhibited a dose-dependent reduction in cell activity after 24 h of treatment. As shown in Figure 8B, within the concentration from 10.0 to 80.0 μg/mL, cell viability in both the MOLP-SeNPs and MOLP treatment groups fell in a dose-dependent manner relative to controls. Notably, the cell viability in the MOLP group prominently came short of that in the MOLP-SeNPs group, suggesting that MOLP-SeNPs exert a stronger cytoprotective effect than MOLP. This is attributed to the low toxicity and antioxidant properties of zero-valent Se. This finding is consistent with previous observations showing that SeNPs chelated with extracellular polysaccharides from *Cordyceps sinensis* enhance biological activity while reducing toxicity [20].

In the concentration from 5.0 to 60.0 μg/mL, the cell survival rate in the MOLP-SeNPs treated group exceeded 90%. Based on these results, MOLP-SeNPs and MOLP at concentrations of 5.0–60.0 μg/mL were selected for in-depth experiments. As exhibited in Figure 8C, relative to controls, incubation with 0.6 mM H_2_O_2_ for 4 h significantly decreased HepG2 cell viability. However, pre-treatment with MOLP-SeNPs or MOLP for 24 h significantly increased cell viability compared to the H_2_O_2_ group, indicating that both MOLP-SeNPs and MOLP were effective in inhibiting oxidative damage. The highest cell viability in these two groups was 95.78% and 90.65%, respectively, which were 1.58 and 1.49 times higher than that of the H_2_O_2_ group (*p* < 0.05). Moreover, the cell survival rate in the MOLP-SeNPs group prominently exceeded that in the MOLP group. This might be due to the combined effect between the polysaccharide and Se after their chelation, resulting in enhanced antioxidant activity and reduced toxicity of MOLP-SeNPs. The results of the cellular experiments indicate that the complex provides a favorable protective effect against oxidative stress at the cellular level.

## 4. Conclusions

Our research presented a simple approach to synthesizing size-controlled SeNPs using MOLP as a stabilizer and dispersant in a sodium selenite-ascorbic acid redox system. Infrared and fluorescence spectra confirmed that selenium bound to functional groups, like C-H, C-O, and -COOH on the surface of MOLP to form new chelates. Particle size, XRD, and SEM analyses revealed that during the chelation of MOLP with selenium, folding and aggregation occurred, resulting in irregular particle shapes, new crystal forms, and a rougher and denser overall appearance. Stability tests showed that MOLP-SeNPs exhibited higher chemical bonding energy, as well as better thermal and structural stability, with enhanced storage stability. Moreover, during the in vitro simulated digestion process, both MOLP and MOLP-SeNPs exhibited enhanced antioxidant capacity. This antioxidant capacity peaked after intestinal digestion, with MOLP-SeNPs demonstrating 1.13 and 1.19 times the ABTS•^+^ and •OH scavenging abilities of MOLP, respectively. Cellular experiments revealed that when MOLP and MOLP-SeNPs were applied to H_2_O_2_-induced HepG2 cells, the highest cell survival rates were 1.58 and 1.49 times that of the H_2_O_2_ group, respectively, indicating that MOLP-SeNPs provided stronger protective effects on H_2_O_2_-induced HepG2 cells. Future studies should focus on exploring the uptake, transport, and regulatory mechanisms of MOLP-SeNPs in vivo to further assess their potential health benefits and industrial applications.

## Figures and Tables

**Table 1:** Response surface optimization level.

Factors	Level		
	−1	0	1
*A* MOLP: Na_2_SeO_3_ (*v*/*v*)	1:2	1:1	4:3
*B* pH	6.0	8.0	10.0
*C* Temperature (°C)	25	35	45

**Table 2:** Response surface test scheme and results.

Number	*A* Volume Ratio (*v*/*v*)	*B* pH	*C* Temperature (°C)	Particle Size (nm)
1	−1	−1	0	321.02 ± 12.32
2	−1	1	0	363.35 ± 16.17
3	−1	0	−1	324.38 ± 13.52
4	−1	0	1	350.26 ± 14.28
5	0	−1	−1	302.18 ± 10.27
6	0	1	−1	293.75 ± 9.63
7	0	−1	1	246.16 ± 12.74
8	0	1	1	410.78 ± 13.62
9	0	0	0	176.73 ± 11.59
10	0	0	0	186.20 ± 10.24
11	0	0	0	202.97 ± 8.47
12	0	0	0	160.32 ± 9.69
13	0	0	0	175.64 ± 12.84
14	1	−1	0	219.06 ± 11.92
15	1	1	0	348.69 ± 10.03
16	1	0	−1	293.35 ± 12.84
17	1	0	1	335.63 ± 13.27

**Table 3:** Analysis of variance.

Source	Sum of Squared Deviations	df	Mean Square	*F*-Value	*p*-Value	Significance
Model	94,987.9	9	10,554.21	44.8	<0.0001	**
*A* Volume ratio	3291.85	1	3291.85	13.97	0.0073	**
*B* pH	13,460.3	1	13,460.3	57.13	0.0001	**
*C* Temperature	2085.61	1	2085.61	8.85	0.0207	*
*AB*	1905.32	1	1905.32	8.09	0.0249	*
*AC*	67.24	1	67.24	0.29	0.6097	
*BC*	7486.58	1	7486.58	31.78	0.0008	**
*A* ^2^	22,237.17	1	22,237.17	94.38	<0.0001	**
*B* ^2^	15,150.44	1	15,150.44	64.3	<0.0001	**
*C* ^2^	22,352.07	1	22,352.07	94.87	<0.0001	**
residual	1649.27	7	235.61			
lost proposal	666.89	3	222.3	0.91	0.5131	ns
pure error	982.37	4	245.59			
aggregate	96,637.16	16				
*R*^2^ = 0.9829 Adj *R*^2^ = 0.9610 Pre *R*^2^ = 0.8737						

Annotation: *p* < 0.01 reaches statistical significance and is denoted by **, *p* < 0.05 holds statistical significance and is denoted by *, and *p* > 0.05 reaches no statistical significance and is expressed by ns.

## Data Availability

The original contributions presented in this study are included in the article. Further inquiries can be directed to the corresponding author.
